# Supplementary material for: StTCTP Positively Regulates StSN2 to Enhance Drought Stress Tolerance in Potato by Scavenging Reactive Oxygen Species
Source: Int J Mol Sci. 2025 Mar 20;26(6):2796. doi: 10.3390/ijms26062796 (PMC11943270; doi:10.3390/ijms26062796)
Supplement: Supplementary file 1 [file ijms-26-02796-s001.zip › Supplementary Figure S1.pdf]

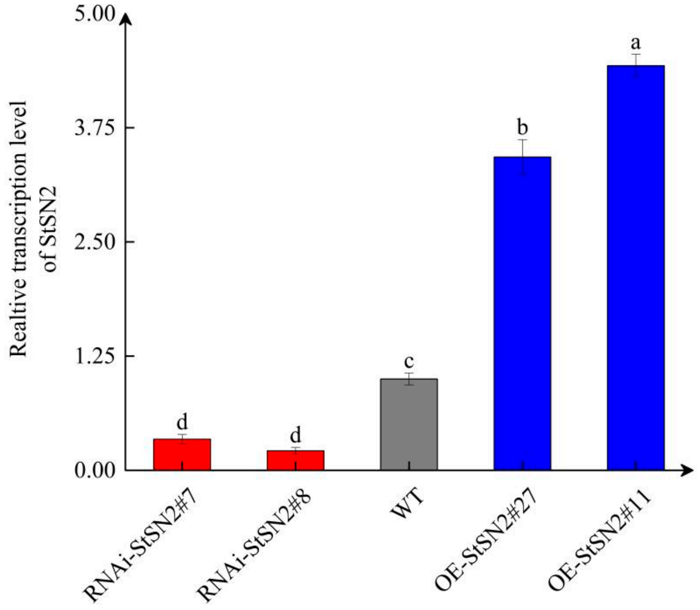

Supplementary Fig. S1 qPCR detection of *StSN2* expression level in the tuber of WT, *RNAi-StSN2* and overexpression lines. Data are shown as means±SD (*n*=3, Student's t-test). Error bars represent standard deviation of the three replications. Different capital letters indicate significant differences(*P*<0.05).
