# Supplementary material for: StTCTP Positively Regulates StSN2 to Enhance Drought Stress Tolerance in Potato by Scavenging Reactive Oxygen Species
Source: Int J Mol Sci. 2025 Mar 20;26(6):2796. doi: 10.3390/ijms26062796 (PMC11943270; doi:10.3390/ijms26062796)
Supplement: Supplementary file 1 [file ijms-26-02796-s001.zip › Supplementary Figure S2.pdf]

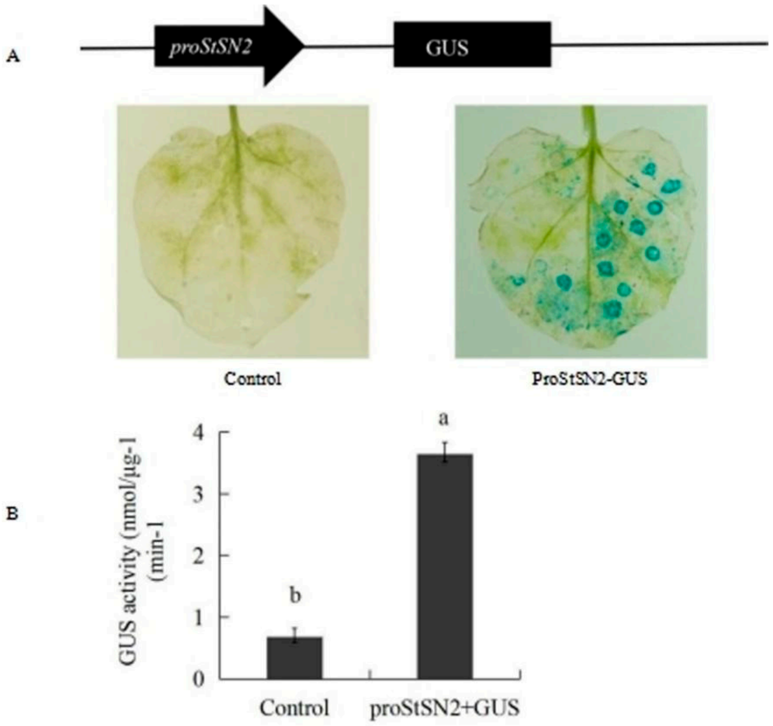

Supplementary Fig. S2 Analysis of β-glucuronidase (GUS) activity driven by promoter fragments of *StSN2.* **A** GUS staining. **B** GUS activity determination.
