# Supplementary material for: StTCTP Positively Regulates StSN2 to Enhance Drought Stress Tolerance in Potato by Scavenging Reactive Oxygen Species
Source: Int J Mol Sci. 2025 Mar 20;26(6):2796. doi: 10.3390/ijms26062796 (PMC11943270; doi:10.3390/ijms26062796)
Supplement: Supplementary file 1 [file ijms-26-02796-s001.zip › Supplementary Figure S3.pdf]

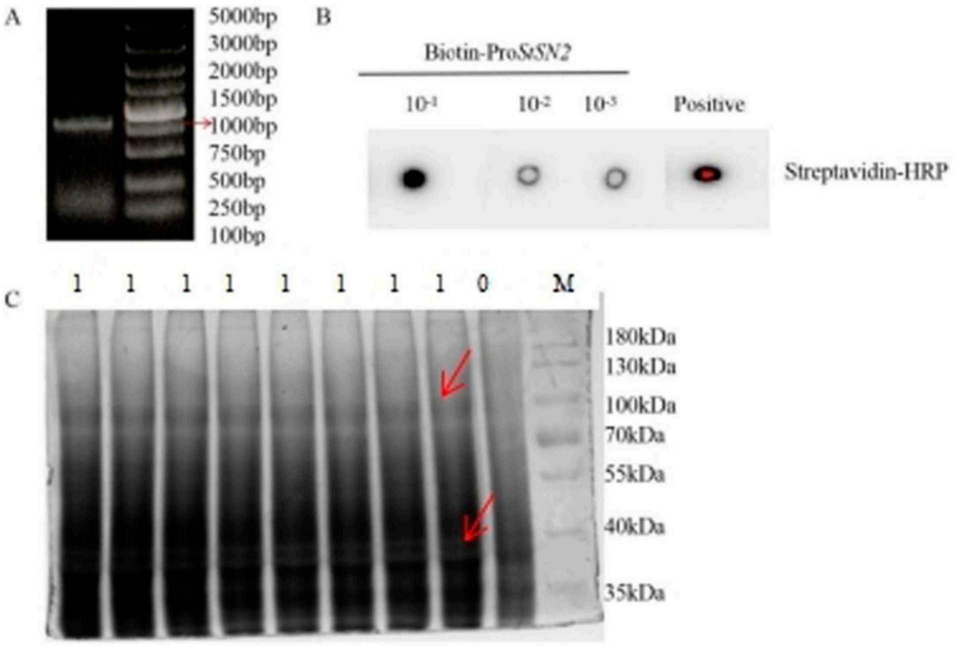

Supplementary Fig. S3 Screening of possible regulators for *ProStS2* based on BAS. **A** Detection of *StSN2* promoter by agarose gel electrophoresis. **B** Immunoblot analysis fo r biotinylated *proStSN2* using streptavidin conjugated with HRP. **C** Silver staining DNA-pu ll-down based on BAS. 0 represents the control group, 1 represents the experimental grou p, M protein marker. Red arrows represent specific protein bands.
