# Supplementary material for: StTCTP Positively Regulates StSN2 to Enhance Drought Stress Tolerance in Potato by Scavenging Reactive Oxygen Species
Source: Int J Mol Sci. 2025 Mar 20;26(6):2796. doi: 10.3390/ijms26062796 (PMC11943270; doi:10.3390/ijms26062796)
Supplement: Supplementary file 1 [file ijms-26-02796-s001.zip › Supplementary Figure S4.pdf]

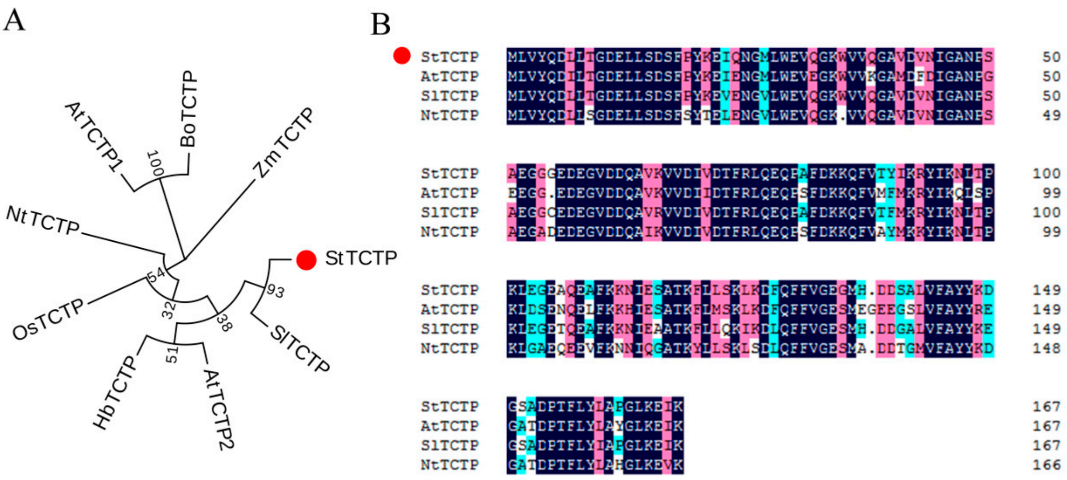

Supplementary Fig. S4 Phylogenetic analysis and amino acid sequence alignment of StTCTP. **A** Phylogenetic tree analysis of Arabidopsis, tobacco, rubber, rice, tomato, corn, and other TCTP and StTCTP. StTCTP is indicated with a red dot. The full-length amino acid sequences were downloaded from NCBI database. **B** Comparison of amino acids in different species of TCTP. StTCTP is indicated with a red dot.
