# Supplementary material for: StTCTP Positively Regulates StSN2 to Enhance Drought Stress Tolerance in Potato by Scavenging Reactive Oxygen Species
Source: Int J Mol Sci. 2025 Mar 20;26(6):2796. doi: 10.3390/ijms26062796 (PMC11943270; doi:10.3390/ijms26062796)
Supplement: Supplementary file 1 [file ijms-26-02796-s001.zip › Supplementary Figure S5.pdf]

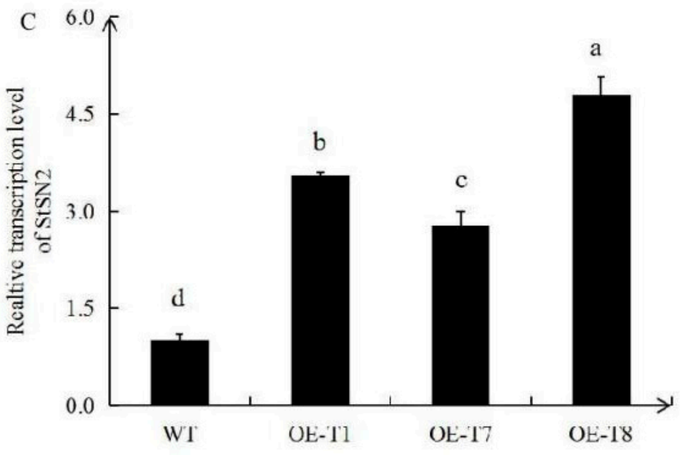

Supplemental Fig. S5 Relative transcript level of *StSN2* in WT and overexpression lines detected by qRT-PCR. Data were normalized using the 2 $^{-\Delta\Delta Ct}$ method and elongation factor 1α (EF-1α) was used as the internal reference for data normalization. The transcript level of *StSN2* in the WT control was sent to 1. Data are shown as means ± SD (n=3, Student's t-test). Error bars represent standard deviation of t hree replicates. Different lowercase letters indicate significant differences (P≤0.05).
