# Supplementary material for: StTCTP Positively Regulates StSN2 to Enhance Drought Stress Tolerance in Potato by Scavenging Reactive Oxygen Species
Source: Int J Mol Sci. 2025 Mar 20;26(6):2796. doi: 10.3390/ijms26062796 (PMC11943270; doi:10.3390/ijms26062796)
Supplement: Supplementary file 1 [file ijms-26-02796-s001.zip › Supplementary File S1.pdf]

Promoter sequences of *StSN2* in the ‘Chuanyu 10’potato cultivar

>Soltu.DM.01G050660

TGTTTTAAAGAGATATACATGAATGGTACTTTGAATTATTTGTTAGATAGAATCTTAAGTT
CTCTTTTGGCCAAGGATAATATAATTAGGAACCAACTTGTTTTAATTAATGTAGCTTTGTA
GACTTTGTGTTAGCTATGTACACCTACCACAGCACTTATATACACTTTGAAAAATGTACTA
ATCATTCCAGACTCCATGATTAAGCTGTTAGATATTTTATCAAGTAATTAATTAAATTCCTT
TTCTCGATTTAATAGTATACTGTTAGGATCGAATTCACACACACTTGATAAATGAAGAATA
CAAGAGTTTTCGAGAGAGATGAAAAATCTAGAGAGAGAAACCAATAGTTTGTGGTAACAC
TTCGTGAATAAACTTTTACAGTGGTGAGATATATATATTAATAATTCAGAGTATGAACAGT
TACAGAGAATAAATACAGAATAAACAGTACAATCTAAAATTTAAACCGCGTAAACATTAA
TATATGTCAGTATATCAAATATTAATCAGGCTTTATAACCTAACATATACCCCATTAACCT
ACTACAGTATTAAGCATCACGTACGAAAAAAAAATCGCATGCATGAAACCTACTTGTATA
GTATATATATATATATATTTTTTTTTTTTTAAAAATATTCTTTTTGCCAAGTGTGTTTTTCT
ATTCTATTTGTCTTCATATACATGATTCTATACATTACCACTATTTCCCCCCACTATAAATA
CCCCATTTACTAATCAATTTTCATAAATCAATTTTTTGGAGAAAAAATCTCAAAAATATTT
CAAATTCCA
