# Supplementary material for: StTCTP Positively Regulates StSN2 to Enhance Drought Stress Tolerance in Potato by Scavenging Reactive Oxygen Species
Source: Int J Mol Sci. 2025 Mar 20;26(6):2796. doi: 10.3390/ijms26062796 (PMC11943270; doi:10.3390/ijms26062796)
Supplement: Supplementary file 1 [file ijms-26-02796-s001.zip › Supplementary Table S1.pdf]

Supplementary Table S1. Quantitative RT-PCR for RNA sequencing validation.

Gene	Purpose	Forward/Reverse
StSN2	qPCR	TAACAGATGTAGCCACTGAC
		ACAACAAGTTCCACATGCCC
Pro-StSN2	Yeast one-kybrid	CGACTCACTATAGGGCGAATTCTGTAGTTGAACTTTTTATCA
		GACCGCGGATCGATTCGCGAACGCGTTGGAATTTGAAATATTTTTCT
StSN2	Luciferase complementary	CTATAGGGCGAATTGGGTACCTGTAGTTGAACTTTTTATCA
		TAGAACTAGTGGATCCCCCGGGTGGAATTTGAAATATTTTTC
StSN2	Kinase assay	GGAGCTCGGTACCCTCGAGGGATCCATGGCCATTTCGAAAGC
		TTAAGCAGAGATTACCTATCTAGATTAAGGGCATTTACGTTTGT
StTCTP	qPCR	TCAAGATCTCCTCACCGGTG
		CATCTTCACCTCCACCCTCA
StTCTP	Yeast one-kybrid	CGACGTACCAGATTACGCTCATATGTTGGTTTATCAAGATCTCCT
		CGATGCCCACCCGGGTGGAATTCTAGCACTTGATCTCCTTCAAG
StTCTP	luciferase complementary	CGCGGTGGCGGCCGCTCTAGAATGTTGGTTTATCAAGATCTCCT
		TCAGCGTACCGAATTGGTACCCTAGCACTTGATCTCCTTCAAG
StSnRK2.2	qPCR	TTATGGAGTACGCAGCAGGT
		CCCACAGTCGACTTTGGTTG
StSnRK2.3	qPCR	TGTATGTCATGCTGGTGGGT
		ACGTTGATCTGGCTCCTCAA
StSnRK2.4	qPCR	TTTTGGCCTCGAATGCAACA
		ACGTTGATCTGGCTCCTCAA
StSnRK2.6	qPCR	GATCGCATCTGTCAAGCTGG
		ACTTGGGACGTGAATGCAAC
