# Supplementary material for: StTCTP Positively Regulates StSN2 to Enhance Drought Stress Tolerance in Potato by Scavenging Reactive Oxygen Species
Source: Int J Mol Sci. 2025 Mar 20;26(6):2796. doi: 10.3390/ijms26062796 (PMC11943270; doi:10.3390/ijms26062796)
Supplement: Supplementary file 1 [file ijms-26-02796-s001.zip › Supplementary Table S2.pdf]

Supplementary Table S2 The identified StTCTP from DNA-pull-down

Accession	Protein names	PepCount	UniquePepCount	CoverPercent	Prorein Coverage (%)	MW	PI
A0A3Q7ENN4	TCTP domain-containing protein	2	1	8.36%	6.08	43745.36	4.54
